# Tuberculosis-Associated MicroRNAs: From Pathogenesis to Disease Biomarkers

**DOI:** 10.3390/cells9102160

**Published:** 2020-09-24

**Authors:** Alessandro Sinigaglia, Elektra Peta, Silvia Riccetti, Seshasailam Venkateswaran, Riccardo Manganelli, Luisa Barzon

**Affiliations:** 1 Department of Molecular Medicine, University of Padova, 35121 Padova, Italy; alessandro.sinigaglia@unipd.it (A.S.); elektra.peta@unipd.it (E.P.); silvia.riccetti@unipd.it (S.R.); riccardo.manganelli@unipd.it (R.M.); 2 School of Chemistry, Kings Buildings, The University of Edinburgh, Edinburgh EH9 3FJ, UK; sesha.venkateswaran@ed.ac.uk

**Keywords:** microRNA, tuberculosis, diagnosis, biomarker, pathogenesis, latent infection, disease progression, response to therapy, innate immunity, apoptosis, autophagy

## Abstract

Tuberculosis (TB) caused by *Mycobacterium tuberculosis* is one of the most lethal infectious diseases with estimates of approximately 1.4 million human deaths in 2018. *M. tuberculosis* has a well-established ability to circumvent the host immune system to ensure its intracellular survival and persistence in the host. Mechanisms include subversion of expression of key microRNAs (miRNAs) involved in the regulation of host innate and adaptive immune response against *M. tuberculosis*. Several studies have reported differential expression of miRNAs during active TB and latent tuberculosis infection (LTBI), suggesting their potential use as biomarkers of disease progression and response to anti-TB therapy. This review focused on the miRNAs involved in TB pathogenesis and on the mechanism through which miRNAs induced during TB modulate cell antimicrobial responses. An attentive study of the recent literature identifies a group of miRNAs, which are differentially expressed in active TB vs. LTBI or vs. treated TB and can be proposed as candidate biomarkers.

## 1. Introduction

Tuberculosis (TB), caused by *Mycobacterium tuberculosis*, is one of the most lethal infectious diseases worldwide [1]. The latest estimates indicate that approximately one fourth of all people worldwide have been infected by *M. tuberculosis* and that TB causes 1.4 million deaths every year [2].

**Figure 1:**
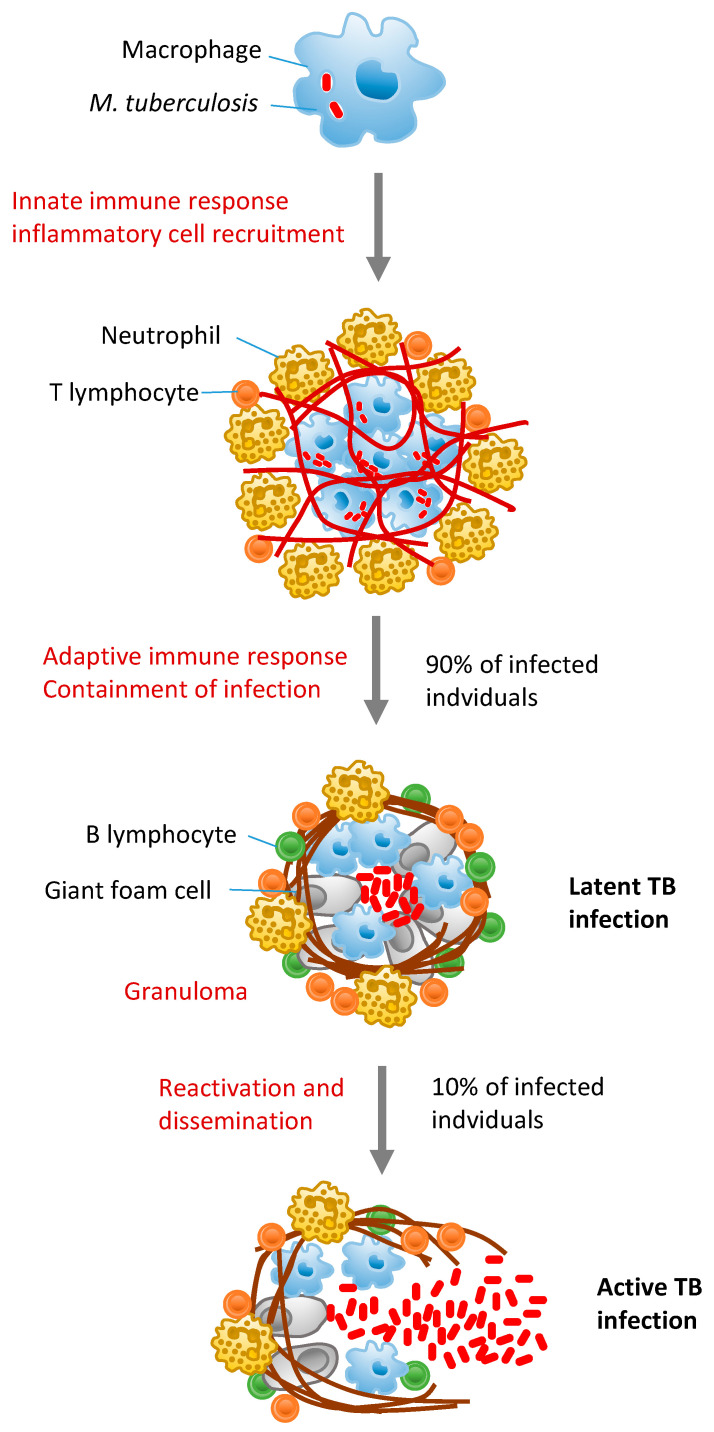
Tuberculosis pathogenesis and disease progression. *Mycobacterium tuberculosis* infection initiates with inhalation of droplets that carry bacteria and their uptake by alveolar macrophages. Innate immune responses characterize the initial phase of infection, with recruitment of inflammatory cells in the lung. Bacterial dissemination to the draining lymph node leads to T cell priming and expansion of antigen-specific T cells. Recruitment of activated macrophages, neutrophils, T cells, and B cells in the lung leads to granuloma formation, which contains *M. tuberculosis* in a latent status. However, in about 10% of infected individuals, *M. tuberculosis* escapes immune control and granulomas are disrupted, with release of infectious bacteria.

A key pathogenic feature of *M. tuberculosis* is its ability to survive for a long time in the human host, inside macrophages in tubercle granulomas [3]. Macrophages are key components of the host innate immune response against *M. tuberculosis* that can eliminate mycobacteria through different mechanism, such as induction of apoptosis, immune-inflammatory responses, and phagocytic activity [4]. However, the pathogen can counteract host antimicrobial mechanisms to ensure survival and persistence. In most *M. tuberculosis* infections, the host immune response is able to arrest bacterial growth and clear the microorganisms or induce a status of latent tuberculosis infection (LTBI). However, about 5–15% of LTBI progress to active TB with pulmonary and/or extra pulmonary involvement [5]. Active TB generally manifests soon after infection, but, in some cases, it may occur even years after primary infection, because of reduced immune response, thus indicating the importance of innate and adaptive immunity in *M. tuberculosis* control [6] (Figure 1).

Bacteria, including *M. tuberculosis*, have evolved several strategies to evade host innate immunity and to survive inside host immune cells. *M. tuberculosis* can modulate several cellular processes such as phagosome–lysosome fusion in macrophages, apoptosis, autophagy, inflammation, innate immune response, MHC class II expression, and antigen presentation by MHC class I in dendritic cells (DCs) [7]. Mechanisms of immune evasion include dysregulation of the host microRNAs (miRNAs) that are involved in control of these biological processes [8,9].

MiRNAs are small non-coding RNAs, typically 18–24 nucleotides in length, which are involved in the regulation of gene expression at post-transcriptional level and influence many biological processes including the immune response. To perform their regulatory functions, miRNAs bind to complementary sequences in the 3′-untranslated region of mRNA targets leading to transcript degradation or translational inhibition [10]. The human genome encodes about two thousands potentially functional miRNAs and each miRNA may suppress multiple genes, while one mRNA can be targeted by multiple miRNAs. Specific anomalies of miRNA expression have been associated with several diseases, including infectious diseases, and investigated as potential diagnostic markers or therapeutic tools [11,12]. Several studies highlighted changes in the levels of circulating miRNAs in patients with TB and identified miRNA signatures that could discriminate between patients with active TB and those with LTBI [13]. The development of new diagnostic and prognostic biomarkers would be particularly useful for screening *M. tuberculosis* infection and disease, for which current methods are still unsatisfactory. In fact, direct diagnosis of *M. tuberculosis* infection by molecular testing and bacterial culture has low sensitivity and long turnaround time, respectively, while indirect diagnosis by tuberculin skin test or interferon gamma (IFNγ) release assay (IGRA) is not able to differentiate LTBI from active TB or to identify patients at risk of disease progression [13,14].

Recent review articles highlighted the relevant role of host miRNAs in the immune response to *M. tuberculosis* infection, their potential as TB biomarkers [15,16] and as targets or tools for therapeutic interventions [17]. However, methodological variability and technical hurdles in miRNA profiling represent important limitations for comparisons of results among studies, as excellently discussed by Ruiz-Tangle and colleagues [18]. With the aim to pursue the effort at identifying potential diagnostic and prognostic miRNA biomarkers, we present here an updated and comprehensive review of basic and clinical studies that identified miRNAs involved in TB pathogenesis, escape from host antimicrobial response, and associated with TB progression or response to anti-TB therapy.

## 2. MicroRNAs in Tuberculosis Pathogenesis

Several studies reported altered expression profiles of circulating and cellular miRNAs in patients with active TB vs. those with LTBI or healthy controls [19]. Differentially expressed miRNAs were further investigated to identify their role within the innate immune response during *M. tuberculosis* infection. In vitro and in vivo experiments confirmed the involvement of miRNAs in modulating gene expression in the major target cells of *M. tuberculosis*, like macrophages, DCs, natural killer (NK) and T cells [20]. *M. tuberculosis* can induce or inhibit miRNA expression in order to escape the immune response. Triggering the apoptotic pathway, induction of autophagy, stimulation of IFNγ and tumor necrosis factor alpha (TNFα) secretion are some of the mechanisms adopted by the host cells during bacterial infection. For example, among miRNAs that are upregulated in TB patients, miR-146a-5p, miR-21-5p, miR-99b-5p and miR-132-5p negatively regulate host inflammatory pathways triggered by Toll-like receptor (TLR) signaling in myeloid cells, thus promoting *M. tuberculosis* survival [21]. Other miRNAs that are upregulated in *M. tuberculosis*-infected macrophages, like miR-27a-5p, miR-33, miR-125-5p and miR-144-5p, inhibit autophagosome formation and *M. tuberculosis* killing by macrophages [22,23]. MiR-29a-3p and miR-125-5p, both upregulated in infected macrophages, directly target IFNγ and TNFα, hence suppressing the immune response to intracellular *M. tuberculosis* [24,25]. Cell apoptosis and inflammasome induction are other mechanisms of defense against intracellular pathogens, which are regulated by *M. tuberculosis*-modulated host miRNAs, like miR-325-3p and miR-20b-5p [26,27]. On the other hand, some miRNAs, like miR-155-5p and let-7f, which are modulated in the course of *M. tuberculosis* infection, play a key role in the activation of host innate and adaptive immunity and clearance of bacteria [28,29,30].

The following paragraphs briefly describe the most studied miRNAs involved in TB pathogenesis and evasion of host innate immunity, as summarized in Table 1.

## 3. Modulation of Innate and Adaptive Immunity

The innate immune response that leads to *M. tuberculosis* infection control initiates with pathogen recognition and uptake by resident lung macrophages, which results in cytokine and chemokine production, expression of immune receptors, and initiation of host defense mechanisms, such as the production of antimicrobial molecules and reactive oxygen species [31]. Recognition of *M. tuberculosis* occurs through different pattern recognition receptors and associated molecules, like TLR2, TLR9, the adaptor molecule MYD88, DC-SIGN, and NLRP3. Stimulation of these pattern recognition receptors induces the expression of pro-inflammatory cytokines, chemokines and cell adhesion receptors that induce immune cell mobilization and activation [32]. Besides lung macrophages, DCs, NK cells, neutrophils and other immune cells contribute to the early innate response against *M. tuberculosis*, which can be effective in preventing infection. However, in most cases, *M. tuberculosis* is able to survive and proliferate in infected macrophages, which require activation by antigen-specific T cells to kill the pathogen. Actually, the role of innate immunity in the early stage of *M. tuberculosis* infection is to establish a suitable condition for induction of adaptive T cell response. The adaptive immune response to *M. tuberculosis* is characterized by production of IFNγ and chemokines by antigen-specific T cells, which facilitate recruitment of further T cells and trigger the phagocytic activity against intracellular bacteria of macrophages [32].

### 3.1. MiR-155-5p Inhibits Host Innate Immunity and Promotes M. tuberculosis Clearance

MiR-155-5p is a key regulatory factor in the innate immunity. Its expression is increased in *M. tuberculosis*-infected macrophages [29] and DCs [30] and in the lungs and liver of mice with *M. tuberculosis* infection [28,33]. Overexpression of miR-155-5p promotes the survival of infected macrophages and intracellular bacterial replication during the early innate response to *M. tuberculosis* infection, but promotes *M. tuberculosis* control during the chronic phase of infection [29,34]. In fact, macrophages from miR-155^−/−^ knock out mice control *M. tuberculosis* growth better than wild type macrophages by increasing cellular apoptosis. Accordingly, after low-dose aerosol infection, miR-155^−/−^ mice exhibit enhanced control of *M. tuberculosis* during the early stage of infection, when macrophage function is critical, and this is associated with increased cellular apoptosis in the lungs. However, during the chronic phase of infection, miR-155^−/−^ mice have a higher load of *M. tuberculosis* and increased inflammatory damage in their lungs and associated mortality than wild-type mice [28,29]. In addition, miR-155^−/−^ mice show a decreased number of antigen-specific CD4+ and CD8+ T cells during chronic *M. tuberculosis* infection and a reduced production of protective cytokines, such as IFNγ and TNFα, by T cells [28,29]. During *M. tuberculosis* infection, both in T cells and in macrophages, upregulation of miR-155-5p directly inhibits expression of SHIP1 [25,29], an inositol phosphatase that promotes cell apoptosis through the modification of the PI3K/Akt signaling pathway. Mir-155-5p expression is also induced in *M. bovis* BCG-infected macrophages via TLR2, NF-κB and JNK signaling pathway [35,36]. In infected macrophages, miR-155-5p inhibits expression of his target SHIP1, leading to increased ROS production [36].

In summary, upregulation of miR-155-5p during *M. tuberculosis* infection, despite inhibiting innate immunity during the early phases of infection, exerts a protective role against TB by promoting the survival of both infected macrophages and DCs, allowing their activation and hence recruitment of T cells, which play a key role in the ultimate control of *M. tuberculosis* through their effector functions [29,30].

### 3.2. MiR-29a-3p Targets IFNγ and Is Downregulated in Experimental Mycobacterial Infection

Production of IFNγ by CD4 T cells is considered a major determinant of TB immunity, although it is not the sole effector mechanisms that contributes to CD4 T cells-mediated protective immunity [37]. During intracellular bacterial infection, miR-29a-3p expression inversely correlates with IFNγ production [38]. In particular, miR-29a-3p is downregulated while IFNγ mRNA levels are higher in CD4+ or CD8+ T cells from *M. bovis* BCG-infected mice than in those from the uninfected controls [34]. Experiments showed that IFNγ mRNA is a direct target of miR-29a-3p and that miR-29a-3p suppresses immune responses to intracellular pathogens by targeting IFNγ [38]. However, data on the association between miR-29a-3p and IFNγ in humans are contrasting. Lower levels of miR-21-5p, miR-26a-5p, miR-29a-3p, and miR-142-3p were found in CD4+ T cells from children with active TB than from those with LTBI [39]. At variance, analysis of *M. tuberculosis*-specific IFNγ-expressing T cells in children with TB versus healthy controls did not show any correlation between miR-29a-3p and IFNγ expression [40]. Moreover, suppression of miR-29a-3p in primary human T cells by antagomirs indicated no effect on IFNγ expression after in vitro activation [39].

### 3.3. Targeting IFNγ Signaling by MiR-26a-5p

Similarly, miR-26a-5p and miR-132-3p can attenuate host immune responses and macrophage activation by IFNγ [41]. These two miRNAs, which are up-regulated upon *M. tuberculosis* infection, directly target the transcriptional coactivator p300, a component of the IFNγ signaling cascade [41]. However, opposite results were obtained by another study [42], which reported decreased miR-26a-5p expression in macrophages infected with *M. tuberculosis* and in the lungs, spleen, and lymph nodes of *M. tuberculosis*-infected mice, while miR-26a-5p overexpression led to reduced *M. tuberculosis* survival in macrophages. MiR-26a-5p downregulation was associated with increased expression of the transcription factor KLF4, which was validated as a new target for miR-26a-5p [42]. KLF4 was demonstrated to drive macrophage polarization towards M2 phenotype, characterized by production of arginase and inhibition of autophagy, and to inhibit trafficking of *M. tuberculosis* to lysosomes [42].

## 4. Suppression of Inflammatory Signaling Pathways

Strong immune responses against *M. tuberculosis* are required for TB control, but they need to be strictly regulated. In fact, excessive inflammation induced by Th1 cells may compromise the acquired immune responses and become detrimental for the host, while a poor inflammatory response may allow uncontrolled bacteria growth [31]. In this context, anti-inflammatory regulatory T cells are an important component of the immune response to *M. tuberculosis* infection, as they counterbalance the protective but pro-inflammatory immune response mediated by Th1 cells [43].

### 4.1. MiR-21-5p, An Anti-Inflammatory MiRNA Upregulated in Mycobacterial Infections

Like in *M. tuberculosis* infection, the spectrum of clinical manifestations of leprosy depends on the interactions between the host immune response and the invading *M. leprae* pathogen at the site of disease. Few skin lesions with involvement of local peripheral nerves characterize the mild form of leprosy, called tuberculoid leprosy, while multiple lesions infiltrating nearly all the skin with extensive peripheral nerve involvement characterize the severe progressive disease, called lepromatous leprosy. Skin lesions of tuberculoid leprosy are characterized by innate and adaptive immune responses mediated by T helper type 1 (Th1) cytokines and macrophages programmed to express the vitamin D–dependent antimicrobial pathway, while the immune response in skin lesions of lepromatous leprosy is typically mediated by Th2 cytokines and macrophages with phagocytic activity [44,45].

Comparative analysis of skin lesions of subjects with lepromatous versus tuberculoid disease identified a set of 13 differentially expressed miRNAs, including the top ranking miR-21-5p, miR-24-5p, and miR-146a-5p with significantly higher expression in lepromatous than in tuberculoid disease [46]. Experimental validation studies showed that miR-21-5p is induced in human monocytes infected with *M. leprae* or treated with phenolic glycolipid-I (PGL-I), a virulence factor from the *M. leprae* envelope [46]. In addition, *M. leprae* infection of macrophages upregulates miR-21-5p at the site of infection [46]. MiR-21-5p and other miRNAs associated with lepromatous disease directly target genes involved in host defense mechanisms, such as *CYP27B1* and *IL1B* of the vitamin D antimicrobial pathway, and, indirectly, induce interleukin 10 (IL-10) production and inhibit antimicrobial gene expression [46]. In addition, miR-21-5p overexpression in monocytes blocks TLR2/TLR1-induced antimicrobial activity against *M. tuberculosis*, while its silencing restores the antimicrobial activity [46].

Expression of miR-21-5p is also upregulated in lung macrophages obtained from mice after vaccination with *M. bovis* BCG and in macrophages and DCs infected ex vivo with *M. bovis* BCG [47]. In this infection model, miR-21-5p suppresses host Th1 response by targeting IL-12 and promotes DC apoptosis by targeting Bcl-2 [47].

A recent study showed that *M. tuberculosis* infection of macrophages is associated with inhibition of host glycolysis [48], and this is dependent on the repression of the phosphofructokinase muscle (PFK-M) isoform by miR-21-5p [48]. At variance, *M. tuberculosis* infection of macrophages obtained from miR-21-5p-deficient mice induces lactate production, suggestive of increased glycolysis [48]. In miR-21-5p-deficient macrophages, bacterial intracellular growth is reduced in comparison with wild type macrophages, and this is paralleled by increased production of pro-inflammatory mediators with anti-bacterial activity [48]. Thus, upregulation of miR-21-5p represents a strategy adopted by mycobacteria to evade the host antimicrobial responses and to ensure intracellular survival and replication. On the other hand, IFNγ production by activated macrophages downregulates miR-21-5p, hence augmenting PFK-M expression and macrophage glycolysis [48].

### 4.2. Let-7 Family, Anti-Inflammatory MiRNAs Downregulated in TB

MiRNAs of the let-7 family are downregulated in *M. tuberculosis* infected macrophages at the transcriptional level in a way dependent on the *M. tuberculosis* early secreted antigenic target 6-kDa protein (ESAT-6), a secreted effector that can modulate host immune responses and promote *M. tuberculosis* escape from the phagosome [49]. Transfection of macrophages with let-7 family mimics showed that let-7f is the most effective in attenuating *M. tuberculosis* survival in macrophages, while treatment with let-7f inhibitor augments *M. tuberculosis* survival [49]. In the context of *M. tuberculosis* infection, the deubiquitinating enzyme A20, also known as TNFα-induced protein 3 (TNFAIP3), is a relevant direct target for let-7f and, accordingly, its expression increases while let-7f levels decrease with progression of *M. tuberculosis* infection in mice [49]. A20 is involved in the maintenance of immune homeostasis as feedback inhibitor of the NF-κB pathway through deubiquitination of TRAF-6 and A20-deficient mice are characterized by severe inflammation and premature death [50]. In *M. tuberculosis*-infected macrophages, A20 reduces the production of pro-inflammatory cytokines, chemokines and NO by decreasing NF-κB activity, thus promoting *M. tuberculosis* survival [49].

### 4.3. MiR-125-5p Suppresses TNFα Production and Autophagy Activation

*M. tuberculosis* up-regulates miR-155-5p and miR-125b-5p expression in macrophages through its envelope component lipomannan [24,25] and ESAT-6 [34,51]. In comparison with the nonvirulent *M. smegmatis*, *M. tuberculosis* induces lower levels of miR-155-5p and higher levels of miR-125b-5p [24]. Since miR-125b-5p directly targets *TNFA* [24,25], the imbalance between these miRNAs leads to lower production of TNFα by macrophages, thereby reducing host inflammatory response [25]. Moreover, miR-125a-3p targets the UV radiation resistance-associated gene *UVRAG*, which plays an essential role in the initiation of autophagy through an association of UVRAG with the Beclin 1/Bcl-2/PI(3)KC3 multiprotein complex [24]. Taken together, these data indicate that miR-125a-3p promotes *M. tuberculosis* immune escape by inhibiting innate host defenses and activation of autophagy [24].

### 4.4. MiR-146a-5p, Upregulated by Mycobacteria, Targets TRAF6 and Decreases NO Production

MiR-146a-5p is upregulated in *M. bovis* BCG-infected macrophages both in vitro and in vivo, in which it promotes mycobacteria survival, without affecting phagocytosis [36,52]. In infected macrophages, miR-146a-5p attenuates the activation of NF-κB and mitogen-activated protein kinases signaling pathways, which in turn suppresses the inducible NO synthase (iNOS) expression and NO generation. Mechanistically, these effects are due to miR-146a-5p direct targeting of TNF receptor-associated factor 6 (TRAF6) [52]. In fact, silencing TRAF6 decreased iNOS expression and NO production in *M. bovis* BCG-infected macrophages, while overexpression of TRAF6 reversed miR-146a-5p-mediated inhibition of NO production and clearance of mycobacteria [52].

### 4.5. MiR-223-3p, Overexpressed in TB, Mitigates Excessive Inflammation

Active *M. tuberculosis* infection induces infiltration of neutrophils and macrophages in the lung where they contribute to local inflammation [20]. MiR-223-3p, which plays a relevant role in myeloid cells biology and is enriched in neutrophils and macrophages [53], is abundantly expressed in blood and lung parenchyma in human and murine TB [54,55,56]. In myeloid cells, miR-223-3p controls NF-κB activity and negatively regulates cytokine release in TB [22,54]. Direct targets of miR-223-3p are represented by the chemokine C-X-C motif ligand 2 (CXCL2), C-C motif ligand 3 (CCL3), and IL-6 [54]. Notably, miR-223-3p knock-out mice fail to control pulmonary TB due to an increased amount of aberrant neutrophils migration and exacerbated inflammation [54]. Thus, miR-223-3p is able to control excessive inflammation in TB by regulating leukocyte chemotaxis and NF-kB activity.

### 4.6. MiR-27b-3p and MiR-99b-5p Prevent Excessive Inflammation in TB

In macrophages, *M. tuberculosis* infection upregulates expression of the miR-23b/miR-27b/miR-24-1 cluster [57]. Among these miRNAs, miR-27b-3p is induced by the TLR-2/MyD88/NF-kB signaling pathway and suppresses the production of pro-inflammatory factors and NF-kB activity, thereby providing a negative feedback loop to prevent excessive inflammation during *M. tuberculosis* infection [58]. In addition, miR-27b-3p increases p53-dependent cell apoptosis and the production of reactive oxygen species, while decreasing bacterial burden [58]. In macrophages, the Bcl-2–associated athanogene 2 (Bag2) was identified as direct target of miR-27b-3p, which can reverse miR-27b-3p–mediated inhibition of the production of pro-inflammatory factors and apoptosis induction [58].

MiR-99b-5p is another miRNA capable of modulating host immunity after *M. tuberculosis* infection by controlling TNFα production. Interestingly, miR-99b-5p is significantly up-regulated in *M. tuberculosis*-infected DCs and macrophages [59]. Inhibition of miR-99b-5p expression leads to reduced bacterial growth in DCs and up-regulation of pro-inflammatory cytokines such as IL-6, IL-12, and IL-1β [59].

### 4.7. MiR-142-3p, An Anti-Inflammatory MiRNA Downregulated in TB

MiR-142-3p, downregulated in CD4 T cells and peripheral blood from TB patients [39] and in *M. bovis*-infected macrophages [60], negatively regulates the expression of inflammatory cytokines, like NF-κB, TNF-α, and IL-6, in part by targeting the *IRAK1* gene [60].

## 5. Inhibition of Phagosome Maturation and Autophagy

Inhibition of phagosome acidification, phagosome–lysosome fusion, ESAT-6 secretion system 1 (ESX1)-dependent escape from the phagosome, and inhibition of autophagy are a key pathogenic mechanisms of *M. tuberculosis* to ensure survival and persistence in the host [61]. *M. tuberculosis* has evolved different virulence mechanisms to evade macrophage killing. For example, the cell wall component lipoarabinomannan suppresses the process of fusion between phagosomes and lysosomes and autophagy. Different miRNAs modulated upon *M. tuberculosis* infection target the autophagy process and phagolysosome maturation.

### 5.1. MiR-33 Locus MiRNAs Target Autophagy Effectors

The miR-33 locus consists of two intronic miRNAs miR-33a-5p and miR-33b-5p and the respective passenger strands miR33a-3p and miR-33-3p, which co-regulate a set of genes involved in cellular cholesterol export and fatty acid oxidation [62,63]. These miRNAs are upregulated in macrophages upon *M. tuberculosis* infection or treatment with mycobacterial cell wall constituents via an NF-κB-dependent mechanism [23]. In infected macrophages, expression of the miR-33 locus contributes to the formation of fatty-acid-rich lipid bodies, which serve as source of nutrients for intracellular mycobacteria and to the inhibition of autophagy pathways, lysosomal function and fatty acid oxidation, though direct targeting of several genes encoding autophagy effectors (such as ATG5, ATG12, LC3B, and LAMP1) [23]. Another target of miR-33a-5p was identified in the kinase AMPK (adenosine 5′ monophosphate-activated protein kinase), which activates the transcription factors FOXO3 and TFEB, both promoting the expression of genes involved in the biogenesis and function of autophagosomes and lysosomes [23]. Silencing of miR-33 in macrophages was demonstrated to enhance *M. tuberculosis* clearance via the autophagy pathway, thus highlighting a mechanism exploited by *M. tuberculosis* to enable intracellular survival and persistence in the host [23].

### 5.2. MiR-27a-5p Downregulates Culcium Signaling and Autophagosome Formation

Expression profiling of miRNAs showed that miR-27a-5p is abundantly expressed in active TB patients, *M. tuberculosis*-infected mice and infected murine primary macrophages, where it promotes bacterial survival through inhibition autophagosome formation [22]. Notably, miR-27a-5p knockout mice infected with *M. tuberculosis* have less histological damage and inflammatory infiltrates in their lungs than wild type mice, thus indicating that miR-27a-5p confers susceptibility to *M. tuberculosis* infection [22]. To investigate the feasibility of miR-27a-5p suppression as a therapeutic strategy for TB, mice were infected with *M. tuberculosis* via the aerosol route and then intraperitoneally injected with a miR-27a-5p antagomir solution. Indeed, treatment resulted in suppression of miR-2a-5p expression in the lung and spleen and significant reduction of bacterial load and tissue damage in the lungs [22]. A key target of miR-27a-5p in TB pathogenesis is CACNA2D3, a component of a voltage-dependent calcium transporter located in the endoplasmic reticulum [22]. Targeting of this transporter leads to the downregulation of Ca^2+^ signaling and, as a consequence, to the inhibition of autophagosome formation and xenophagy, hence promoting the intracellular survival of *M. tuberculosis* [22].

### 5.3. MiR-144-5p Inhibits Phagosome Maturation and T Cell Function

MiR-144-5p is overexpressed in the blood, PBMCs, and sputum of active TB patients and its levels decrease after anti-tuberculosis therapy [64,65]. In vitro, it is upregulated in human monocyte-derived macrophages after *M. tuberculosis* infection [66]. MiR-144-3p directly binds the 3′UTR region of *DRAM2* mRNA (DNA damage regulated autophagy modulator 2), which encodes a transmembrane lysosomal protein that interacts with key components of the autophagy machinery [21]. In PBMCs of patients with active TB, miR-144-5p is mainly expressed by T cells. Forced miR-144-5p overexpression in T cells decreases cell proliferation and reduces IFNγ and TNFα secretion upon TCR stimulation [65]. All together, these data suggest that miR-144-5p upregulation in macrophages and T cells during *M. tuberculosis* infection represents another mechanism to circumvent anti-tuberculosis immunity through inhibition of phagosome maturation and T cell function.

### 5.4. MiR-155-5p Promotes M. tuberculosis Killing Through Autophagy

MiR-155-5p inhibits the survival of intracellular mycobacteria through different mechanisms, including promoting phagosome maturation and autophagy induction [33]. In infected macrophages, miR-155-5p targets the negative regulator of autophagy Rheb (Ras homologue enriched in brain), which inhibits autophagy via mTOR [33]. At variance, in *M. tuberculosis*-infected DCs, miR-155-5p targets ATG3, an E2-ubiquitin-like-conjugating enzyme with an essential role in autophagosome formation, thereby suppressing autophagy in DCs [30].

### 5.5. MiR-889-5p, Overexpressed in LTBI, Inhibits Autophagy

MiRNA profiling in PBMCs from rheumatoid arthritis patients with LTBI identified miR-889-5p as the top ranking overexpressed miRNA compared with patients without infection [67]. The levels of circulating miR-889-5p were confirmed to be significantly higher in patients with LTBI and to decrease after prophylactic therapy [67]. MiR-889-5p directly targets the cytokine TWEAK (TNF-like weak inducer of apoptosis), whose expression increases in macrophages and PBMCs upon infection with *M. tuberculosis* or exposure to heat-killed *M. tuberculosis* [67]. An in vitro model of human TB granuloma showed TWEAK upregulation during the early phase of infection, followed by a decline an increased expression of miR-889-5p with the development of a granuloma-like structure, representative of a LTBI status [67]. Upon entry to latency, elevated miR-889-5p levels are associated with TNFα and granuloma formation/maintenance [67]. In macrophages, TWEAK induces autophagy and promotes autophagosome maturation through activation of AMP-activated protein kinase. In macrophages infected with *M. bovis* BCG, miR-889-5p overexpression inhibits autophagy and maintains mycobacterial survival in granulomas. Treatment of these granuloma-like structures with adalimumab, an anti-TNF-α monoclonal antibody, reduces levels of both TNF-α and miR-889-5p and causes granuloma destruction and LTBI reactivation [67]. As expected, TWEAK levels are low in LTBI patients and increased after prophylactic therapy [67]. Interestingly, in one LTBI patient receiving adalimumab therapy, circulating TWEAK levels increased at the time of LTBI reactivation and returned to baseline values after anti-TB therapy [67].

## 6. Subversion of Macrophage Death Pathways

Apoptosis of infected macrophages is a strong innate host defense tool against intracellular pathogens, including mycobacteria, as apoptotic vesicles containing bacterial antigens are taken by other phagocytes or DCs. Then, DCs can efficiently present bacterial antigens to naïve T cells, leading to their activation. Virulent *M. tuberculosis* inhibits apoptosis and triggers necrosis of host macrophages to evade bacterial killing and innate immunity and to delay the initiation of adaptive immunity [68]. Necrosis is induced when *M. tuberculosis* escapes from the phagosome and diffuses into the cytosol of macrophages. Necrosis leads to cell lysis and extracellular dissemination of mycobacteria, which can infect other macrophages that have been recruited to the lung. Pyroptosis, a form of necrosis that requires caspase 1 and inflammasome activation, occurs in infected macrophages, when the plasma membrane is damaged by *M. tuberculosis* ESX-1 secretion system or after phagosome rupture. This damage causes activation of NLRP3-dependent IL-1β release and pyroptosis, which facilitates the spread of bacteria to neighboring cells [69].

### 6.1. MiR-20b-5p, Downregulated in M. tuberculosis Infection, Inhibits Inflammasome Activation and Promotes Apoptosis

Macrophages isolated from TB patients have decreased miR-20b-5p level, while NLRP3 inflammasome, a central regulator in the inflammatory process and pyroptosis, is activated [27]. In particular, low miR-20b-5p expression and activated NLRP3/caspase-1/IL-1β pathway were observed in a TB mouse model stably infected with *M. tuberculosis* [27]. In these mice, intravenous injection of miR-20b-5p mimic deactivated the NLRP3/caspase-1/IL-1β pathway and alleviated the inflammatory response. Moreover, transfection of miR-20b-5p in macrophages from TB mice induced M1 to M2 polarization via the NLRP3/caspase-1/IL-1β pathway [27]. MiR-20b-5p resulted downregulated also in a murine macrophage cell line infected by *M. tuberculosis* [70]. In vitro transfection of these macrophages with miR-20b-5p mimics and a miR-20b-5p inhibitor demonstrated that inhibition of miR-20b-5p promotes *M. tuberculosis* intracellular survival through attenuation of cell apoptosis [70]. The mechanisms of inflammasome inhibition and apoptosis induction by miR-20b-5p are conceivably the direct targeting of *NLRP3* [27] and Mcl-1 [70], a negative regulator of cell apoptosis, respectively.

### 6.2. MiR-325-3p, Upregulated in M. tuberculosis Infection, Inhibits Apoptosis

MiR-325-3p is upregulated in experimental *M. tuberculosis* infection in mice and macrophages and in patients with LTBI [26]. MiR-325-3p upregulation occurs also after exposure of macrophages to gamma-irradiated *M. tuberculosis* but not after infection with *M. bovis* BCG, which lacks the RD1 genomic region of pathogenicity [26]. Macrophages with silenced miR-325-3p and miR-325-deficient mice show resistance to *M. tuberculosis,* thus indicating that miR-325-3p promotes *M. tuberculosis* persistence and latency in the host. MiR-325-3p directly targets *LNX1*, which encodes an E3 ubiquitin ligase of the serine/threonine protein kinase NEK6. In macrophages, *M. tuberculosis* leads to LNX1 downregulation and hence NEK6 accumulation, which in turn inhibits apoptosis through activation of STAT3 signaling [26]. Conversely, suppression of the NEK6/STAT3 pathway reduces bacterial burden and improves survival of *M. tuberculosis* infected mice [26].

### 6.3. MiR-155-5p Modulates Apoptosis

MiR-155-5p role in the modulation of apoptosis is controversial. In fact, validated targets of miR-155-5p in *M. tuberculosis*-infected macrophages include: PKI-α, a negative regulator of PKA signaling, whose activation triggers pro-apoptotic genes [35]; the suppressor of cytokine signaling-1 (*SOCS1*), whose suppression leads to increased IL-6 and TNFα production and apoptosis induction [51]; and forkhead box O3 (*FOXO3*), which is involved in cell cycle regulation, innate immune response, and resistance to cell apoptosis [71].

**Figure 2:**
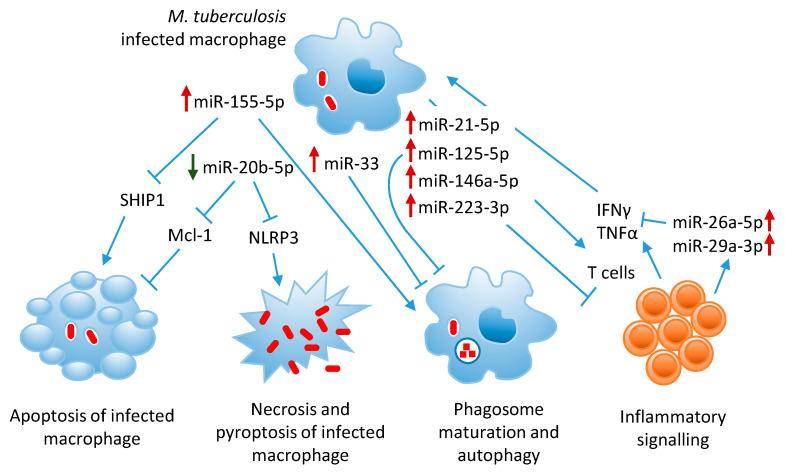
Graphical representation of miRNA regulation of host immune responses against *M. tuberculosis* infection. MicroRNAs that are up-regulated or down-regulated during *M. tuberculosis* infection are indicated with red and green arrows, respectively.

The role of the most relevant miRNAs involved in TB pathogenesis is summarized in Figure 2.

## 7. Biomarker Discovery Studies

Diagnosis of *M. tuberculosis* infection and the distinction between active TB and LTBI remain challenging. In addition, currently available tests do not allow predicting which cases of LTBI have higher risk to progress to overt disease during their lifetime. Approaches for the diagnosis of TB include screening tests, based on the evaluation of host immunity, for detecting *M. tuberculosis* infection in wide populations and more accurate methods, based on direct pathogen detection in clinical samples, for the identification and monitoring of subjects with active TB. A correct strategic approach in the use of screening methods is crucial for both efficiency and sustainability of downstream diagnostics, and for the administration of preventive therapies especially for population groups in whom direct detection of *M. tuberculosis* in blood or sputum might be problematic (i.e., in children) or in settings where excessively elaborate or expensive methods are hardly sustainable. To date, screening methods rely mainly on tuberculin skin tests and/or IGRA, both lacking the ability to distinguish between active TB or LTBI. Since only about 10% of LTBI subjects will develop active disease, the predictive power of such tests may not be optimal. Screening methods based on disease biomarkers (typically quantified in blood samples) could be of help in detecting subjects with higher probability of progressing into active TB, or who are in early stages of disease [72,73].

Aiming to meet these requirements, several clinical studies have identified molecular signatures, generally applied to PCR assays in blood, which have proven to be effective when tested on prospective cohorts. Such studies make use of gene (or protein) expression screening methods (microarrays, RT-PCR arrays, RNA-sequencing) to detect consistent alterations associated with specific clinical features, typically comparing groups of TB patients with LTBI or healthy subjects, and validating a short-list of selected candidate markers in follow-up studies of TB-exposed groups to predict which subjects will develop pulmonary disease. Many different signatures have been proposed, showing higher sensitivity and specificity than the currently used screening tests [74,75,76,77,78,79,80,81]. Moreover, with a similar approach, signatures of genes or proteins whose expression varies in response to TB therapy have been proposed to provide help in follow-up clinical evaluation of treated patients, or to support prognostic evaluation of newly diagnosed TB cases [74,82].

As for cancer and other diseases, miRNAs have been taken into consideration as a possible source for such biomarkers, and many discovery studies have been published, either as screenings in subjects-vs-controls cohorts or aiming at validating miRNAs identified in experimental studies [83].

As overviewed in the previous paragraphs, *M. tuberculosis* infection can cause a dysregulation in the expression of genes (including miRNAs) involved in several physiological pathways (immunity, inflammation, autophagy and apoptosis) which could be potentially used as diagnostic and prognostic biomarkers of disease and response to therapy. Several research groups have tried to identify well-defined miRNA signatures that could be analyzed in easily accessible specimens, with particular focus on circulating miRNAs, which are detected in blood (PBMCs or serum/plasma). The desirable miRNA signature should specifically identify subjects with *M. tuberculosis* infection, and possibly distinguish an active infection (i.e., pulmonary and/or extra-pulmonary tuberculosis) from LTBI. In the next paragraphs, some relevant screening studies performed on serum/plasma or blood cells with different design strategies are briefly reviewed. Data from these and other studies published in the last 10 years are summarized in Table 2 (studies on whole blood, plasma or serum) and in Table 3 (studies on blood cells).

### 7.1. Biomarkers of Active Tuberculosis

The starting step in the definition of miRNAs as effective biomarkers in TB diagnosis is the establishment of a consistent signature of differential miRNA expression in samples from active TB cases compared to healthy controls. During the last decade, several studies with this design have been performed to identify TB-associated miRNA signatures in serum/plasma and blood cells (Table 2 and Table 3). Though serum and plasma are usually preferred as starting material for diagnostic screenings, many researchers used cells (PBMCs or specific cell subsets) for discovery studies on miRNA expression. Studies that performed miRNA profiling by broad-spectrum unbiased methods, like miRNA microarray analysis or small RNA sequencing by next-generation sequencing techniques, should generate more reliable results than studies based on the investigation of a panel of candidate miRNAs. However, screenings by small RNA-sequencing or miRNA microarrays have been generally applied to relatively small groups of subjects (usually less than 10 subjects), and then the identified candidates were validated by targeted assays, like qRT-PCR, in larger groups of subjects. This approach might present limitations for the robustness and reproducibility of results. At variance, several other studies performed only qRT-PCR to validate candidate miRNA biomarkers identified from available datasets or based on the role of the candidate miRNA in TB pathogenesis [111,113,114,117,119].

Unfortunately, poor consensus arises from the results of these screenings, as very few miRNAs are present in the proposed signature of two or more studies. Probably this is due to the different screening methods (various RNA-sequencing and microarrays platforms) or to heterogeneity of cohorts (including size and ethnicity). RNA isolation methods can also influence heavily the results, as is the case of studies that were performed with protocols that specifically recover RNA from blood exosome vesicles [87,88]. Since many miRNAs are abundant in exosomes, this approach can be of particular interest, although the results obtained by the two studies on exosome vesicles-associated miRNAs do not agree [87,88].

Nonetheless, notwithstanding the heterogeneity of the results, some miRNAs have been recurrently identified as candidate biomarkers of TB in more than one study. Among these candidate miRNAs, miR-26a-5p, and miR-29a-3p were identified by different studies as significantly overexpressed in patients with active TB vs. healthy controls [86,88,90,108,109,115]. These are relevant miRNAs in TB pathogenesis, since, as previously discussed, both directly or indirectly target IFNγ, thus suppressing host innate and adaptive immune response against intracellular pathogens. Other anti-inflammatory miRNA, miR-21-5p, and miR-146a-5p, which are overexpressed in active TB patients, are promising diagnostic biomarker to differentiate between active TB and latent infection or an healthy condition [87,92,101]. MiR-155-5p, overexpressed in TB patients and candidate biomarker of active TB, plays a key role in host defense against *M. tuberculosis* [86,96,112].

### 7.2. MiRNAs in Latent vs. Active Tuberculosis

Since the currently available tests do not effectively discriminate between LTBI and active TB, a biomarker with a significant difference in expression between the two conditions would be very useful. Several studies of miRNA expression profiling in serum/plasma [86,90,97,102] or in blood cells [33,39,109,116,118] have been performed in cohorts which included subjects with LTBI along with active TB infection and healthy controls. In some of these studies, significant miRNA signatures were identified that could actually discriminate active TB from LTBI. A study on PBMCs used miRNA-specific microarrays to obtain a short-list of candidates that were validated by qRT-PCR [33]. Interestingly, five miRNAs (miR-424-5p, miR-365a-3p, miR-144-3p, miR-223-3p, and miR-451a-5p) with high expression in PBMCs were upregulated in active TB vs. LTBI, with target predictions suggesting a possible role in hematopoiesis. The same experimental setup was used in another study that identified overexpression of miR-194-5p, miR-21-5p, miR-29c-3p and the downregulation of miR-150-5p in the active TB group compared with both LTBI and HC groups [100]. By using qRT-PCR, miR-29a-3p overexpression was confirmed to be a valuable candidate biomarker to discriminate between active TB and LTBI in a cohort of subjects from Cameroon [86]. In this cohort, expression levels of miRNAs was similar in subjects co-infected with HIV and in those without HIV infection [86]. A recent study in China, which analyzed miRNA expression profile by RNA-sequencing in serum exosomes, found overexpression of miR-1246, miR-2110, miR-370-3p, miR-28-3p, miR-193b-5p and downregulation of miR-3675-5p in active TB, but also identified a set of miRNAs exclusively expressed in latent TB [85]. No validation experiments in a second cohort of patients were performed.

### 7.3. Prognostic Biomarkers of Risk of Progression to Tuberculosis and Response to Therapy

Prospective studies have been performed with the aim to identify prognostic miRNA signatures associated with the risk of progression from LTBI to active TB or predictive of response to anti-TB therapies.

As an example of the first aim, a recent study [91] identified in a cohort of subjects in South Africa and Uganda, developing active TB in a 2 years follow up, a significant miRNA signature of disease-progression (i.e., overexpression of miR-21-5p, miR-484 and downregulation of miR-148b-3p).

As for the second aim, the identification of a miRNA signature with diagnostic value can help in evaluating follow-up subjects during treatment. Theoretical expectations are that miRNAs deregulated at diagnosis can revert to baseline values if therapy is successful. A study in an Indian cohort [95] showed that miR-16-5p and miR-155-5p worked as diagnostic biomarkers in patients with acute TB, while levels were low in healthy controls and in TB patients who successfully completed therapy. These miRNA, though, were selected on literature basis and not after an experimental screening. Other miRNAs (miR-99b-5p, miR-29a-3p, miR-26a-5p) were identified as being downregulated in subjects undergoing successful TB treatment in a large Chinese cohort tested by two different qRT-PCR protocols [90]. At variance, another Chinese study with a similar design and therapeutic regimen, but with a different screening method (i.e., small RNA-sequencing) obtained different results. In this case, treated subjects had downregulated miR-21-5p, miR-92a-3p, miR-148b-3p, and overexpressed miR-125a-5p [92]. Possibly, the reason of such heterogeneous reports could be due to differences in screening methods, thus emphasizing the importance of standardization of protocols to provide more consistent findings. A recent report from Brazil [84], which used small RNA-seq of whole blood from subjects with active TB, latent TB and isoniazide-treated latent TB, identified three miRNAs (let-7a-5p, miR-196b-5p, miR-589-5p) and the small nucleolar RNA SNORD104 as a highly sensitive (100%) classifier to discriminate TB from non-TB groups. However, no highly accurate biomarker were found for the discrimination between TB and LTBI [84]. Interestingly, expression of the four small RNAs changed as expected in a LTBI patient who progressed to active TB (i.e., downregulation for let-7a-5p, miR-196b-5p, and SNORD104, but upregulation for miR-589-5p) and tended to normalize in two patients with active TB for whom whole blood samples were available after completion of therapy [84]. In addition, these small RNAs had similar expression profiles in peripheral whole blood in active TB patients and in PBMCs infected ex vivo with *M. tuberculosis* [84].

Another study investigated the levels of circulating small RNAs in a group of 34 patients with active TB (including 17 with HIV-1 co-infection) who were treated with standard 6-month drug regimen for pulmonary TB [99]. Comparison of small RNA levels before and after completion of therapy showed a significant decrease of plasma small RNA levels after effective treatment, which was independent of HIV-1 co-infection [99]. No single miRNAs nor combination of small RNAs were significantly associated with successful TB treatment, even though there was a trend towards a decrease of miR-17-3p, miR-29a-3p, miR-133a, and SNORD61 in those who responded to therapy (*n* = 30) compared with those who did not (*n* = 4) [99].

## 8. Conclusions

Tuberculosis remains one of the most relevant health emergencies worldwide, with estimates of 1.4 million fatalities in 2018. Despite great advances in diagnosis and therapy, the large proportion of infected subjects in populations, especially in low-income countries, requires the development of new screening methods capable of detecting high-risk conditions with easily manageable assays.

With this aim, the search for ideal biomarkers for detection in blood (or other non-invasive samples) has found miRNAs as potentially ideal candidates. MiRNAs are indeed strictly involved in the pathogenesis pathways of TB, and in particular in the regulation of immune responses related to the switch between latent and active infection. Moreover, many of them are present in detectable amounts in plasma or serum, and can easily be quantified by PCR-based methods.

**Figure 3:**
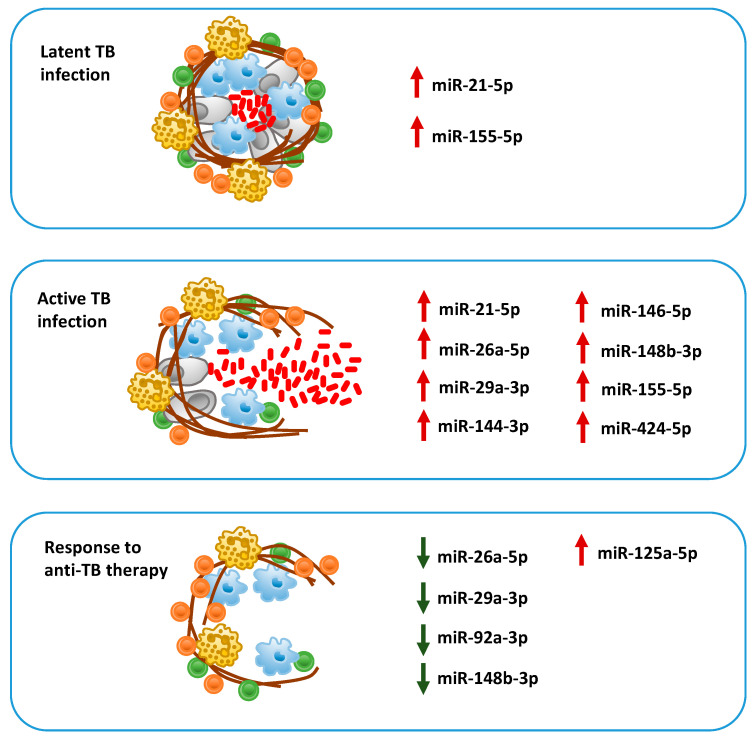
Graphical representation of circulating miRNAs, which are significantly up-regulated (red arrow) or down-regulated (green arrow) in subjects with latent tuberculosis (TB) infection, active TB, or who responded to anti-TB therapy, and have been proposed as candidate biomarkers.

Several studies in recent years have focused on selecting short-lists (signatures) of miRNAs whose expression in consistently related to the development of active TB or to a differential response to therapies. Their common aim is introducing in diagnostic routine screening assays with significant predictive power, improving the accuracy of currently used assays based mainly on tuberculin skin test of IGRA. On the other hand, many research groups are working to establish functional relations between miRNAs expression in differential conditions and their actual biological effect, molecular biology and bioinformatics methods to validate their biological targets and understand their role in TB pathogenesis. This review, in particular, identified some promising candidates, like miR-155-5p, miR-146a-5p, miR-26a-5p, miR-29a-3p, mir-21-5p, mir-144-3p, and miR-424-5p, which can discriminate among active TB from LTBI and healthy condition and predict response to anti-TB therapy (Figure 3). Taken together, these two arms of the general effort of the scientific community are expected to provide in the next future a new generation of screening assays based on miRNAs. These diagnostic tools need to adhere to quality requirements of specificity and sensitivity, but as well to have biological relevance in TB pathogenesis.

## Figures and Tables

**Table 1:** Summary of the role of microRNAs (miRNAs) in tuberculosis (TB) pathogenesis.

Function	Upregulated in TB	Dowregulated in TB
Inhibition of innate immunity	miR-26-5p, miR-132-3p, miR-155-5p	miR-29-3p
Suppression of inflammation	miR-21-5p, miR-27b-3p, miR-99b-5p, miR-125-5p, miR-146a-5p, miR-223-3p	let-7f, miR-20b-5p miR-142-3p
Inhibition of phagosome maturation and autophagy	miR-33 locus, miR-27a-5p, miR-144-5p, miR-889-5p	
Apoptosis inhibition	miR-155-5p, miR-325-3p	

**Table 2:** Summary of studies on miRNA biomarker discovery in blood samples from TB subjects and controls.

Cases No. (Category)	Controls No. (Category)	Country	Samples	Method for Screening	Method for Validation	Up-Regulated in TB	Down-Regulated in TB	Ref.	Year
8 (TB), 21 (LTBI)	6 treated TB, 14 HC	Brazil	blood	RNA-Seq (Illumina)	qRT-PCR	miR-589-5p	miR-196b-5p, let-7a-5p	[84]	2020
60 (TB), 60 (LTBI)	60 HC	China	exosomes from serum	RNA-Seq (Illumina)	qRT-PCR	miR-1246, miR-2110, miR-370-3p, miR-28-3p, miR-193b-5p	miR-3675-5p	[85]	2019
84 (TB), 35 (LTBI)	42 HC	Cameroon	plasma	literature (miRNA selection)	qRT-PCR	miR-29a-3p, miR-361-5p (vs LTBI); miR-155-5p (vs HC)		[86]	2019
15 (TB), 22 (extra-pulmonary TB)	15 HC	India	serum	RNA-Seq (Ion Torrent)	qRT-PCR	miR-146a-5p (TB), miR-125b-5p (EPTB)		[87]	2019
246 (TB)	105 HC	China	exosomes from plasma	Microarray (Affymetrix)	qRT-PCR	miR-20a-5p, miR-20b-5p, miR-26a-5p, miR-106a-5p, miR-191-5p, miR-486-5p		[88]	2019
25 (TB)	25 HC	Iran	exosomes from serum	literature (miRNA selection)	qRT-PCR	miR-484, miR-425-5p, miR-96-3p		[89]	2019
100 (TB)	89 treated TB, 100 HC	China	plasma	miRNA PCR panel (Exiqon)	qRT-PCR	miR-29a-3p, miR-99b-5p (vs HC), miR-29a-3p, miR-99b-5p, miR-26a-5p (vs treated)	miR-21-5p, miR-146a-5p, miR-652-5p	[90]	2018
54 (TB)	54 HC	South Africa, Uganda	serum	qRT-PCR	qRT-PCR	miR-21-5p, miR-484	miR-148b-3p	[91]	2018
53 (TB)	53 treated TB, 53 HC	China	serum	RNA-Seq (Illumina)	qRT-PCR	miR-21-5p, miR-92a-3p, miR-148b-3p (vs treated)	miR-125a-5p (vs treated)	[92]	2017
178 (TB)	95 HC	China	plasma	RNA-Seq (Illumina)	qRT-PCR		miR-22-3p, miR-320a-5p, miR-769-5p	[93]	2017
60 (TB), 32 (MDR-TB)	60 HC	China	serum	RNA-Seq (Illumina)	qRT-PCR	miR-424-5p, miR-4433b-5p (MDR vs. DS); miR-199b-5p, miR-424-5p (vs HC)		[94]	2016
124 (TB)	117 HC	China	serum and sputum	literature (miRNA selection)	qRT-PCR	miR-144-3p		[64]	2016
30 (TB), 19 (MDR-TB)	10 treated TB, 30 HC	India	serum	literature (miRNA selection)	qRT-PCR		miR-16-5p, miR-155-5p	[95]	2016
73 (TB)	69 HC	China	blood	available microarray dataset	none	miR-132-3p, miR-155-5p		[96]	2016
10 (TB), 13 (LTBI)	11 HC	China	plasma	Microarray (Agilent)	qRT-PCR	let-7b-5p, miR-30b-5p		[97]	2016
11 (TB)	10 HC	China	serum	available microarray dataset	miRNA PCR panel (TaqMan)	miR-1249-5p	list of 11 miRNAs	[98]	2015
34 (TB, 17 HIV co-infected)	30 treated TB (14 HIV co-infected)	South Africa	plasma	miRNA PCR panel (MIHS-106Z arrays)	qRT-PCR		miR-29a-3p, miR-17-3p, miR-133a	[99]	2015
17 (TB), 17 (LTBI)	16 HC	Spain	blood	Microarray (Agilent)	qRT-PCR	miR-194-5p, miR-21-5p, miR-29c-3p (vs HC and LTBI)	miR-150-5p (vs HC and LTBI)	[100]	2015
110 (TB)	48 HC	China	serum	literature (miRNA selection)	qRT-PCR	miR-183-5p		[101]	2015
15 (TB), 14 (LTBI)	68 HC	China	serum	RNA-Seq (Illumina)	qRT-PCR	miR-196b-5p, miR-376c-3p		[102]	2014
29 (TB)	37 HC	Egypt	serum	literature (miRNA selection)	miRNA PCR Panel (miScript)	miR-197-3p		[103]	2013
108 (TB)	88 HC	China	serum	RNA-Seq (Illumina)	qRT-PCR	miR-378a-5p, miR-483-5p, miR-22-3p, miR-29c-3p	miR-101-3p, miR-320b	[104]	2013
269 (TB, 73 HIV co-infected), 109 (LTBI)	105 HC	Italy, Tanzania, Uganda	serum	miRNA PCR panel (TaqMan), pooling of samples	qRT-PCR in a subset of individual samples	list of 12 miRNAs (e.g., miR-148a, miR-192, miR-193a-5p, miR-451, miR-590-5p, miR-885-5p)	let-7e-5p	[105]	2013
8 (TB)	8 HC	Germany	serum	Microarray (Agilent)	none	list of 17 miRNAs	miR-574-5p, miR-768-3p, miR-940	[106]	2012
30 (TB)	65 HC	China	serum	miRNA PCR panels (TaqMan)	qRT-PCR	miR-361-5p, miR-889, miR-576-3p, miR-210, miR-26a-5p, miR-432-5p, miR-134		[107]	2012
75 (TB)	55 HC	China	serum	Microarray (Exiqon)	qRT-PCR	miR-93-3p, miR-29a-3p	miR-3125	[108]	2011

**Table 3:** Summary of miRNA biomarker discovery studies in blood cells (in vivo or ex vivo) from TB subjects and controls.

Subjects No. (Category)	Controls No. (Category)	Country	Samples	Method for Screening	Method for Validation	Up-Regulated in TB	Down-Regulated in TB	Ref.	Year
30 (TB), 35 (LTBI)	35 HC	China	PBMCs	available microarray dataset	qRT-PCR	miR-212-3p		[109]	2019
3 (TB)	3 HC	China	PBMCs	small RNA-seq	none	list of 18 miRNAs	list of 23 miRNAs	[110]	2018
12 (TB)	12 HC	South Africa	PBMCs	literature (miRNA selection)	qRT-PCR	miR-320a-3p, miR-204-5p, miR-331-3p, miR-147b, miR-210-3p	miR-197-3p, miR-99b-5p, miR-191-5p	[111]	2018
21 (TB)	21 treated TB	Mexico	PBMCs	literature (miRNA selection)	qRT-PCR	miR-29a-3p, miR-326		[112]	2017
9 (TB)	9 HC	Argentina	PBMCs	literature (miRNA selection)	qRT-PCR		miR-29a-3p, miR-30c-5p, miR-181a-5p, miR-181b-5p	[113]	2017
122 (TB)	130 HC	China	PBMCs, CSF	literature (miRNA selection)	qRT-PCR	miR-29a-3p		[114]	2017
28 (TB)	24 HC	China	PBMCs	Microarray (Agilent)	qRT-PCR	miR-29b-3p	miR-1-3p, miR-155-5p, miR-31-5p, miR-146a-5p, miR-10a-5p, miR-125b-5p, miR-150-5p	[115]	2016
3 (TB), 4 (LTBI)	3 HC	Hong Kong-China	Macrophages ex vivo	miRNA PCR panel (TaqMan)	none	miR-16-5p, miR-137, miR-140-3p, miR-193a-3p, miR-501-5p, miR-598	miR-95	[116]	2015
65 (TB)	60 HC	China	PBMCs	literature (miRNA selection)	qRT-PCR		miR-31	[117]	2015
30 (TB), 28 (LTBI)	30 HC	China	CD4 + T cells	Microarray (Exiqon)	qRT-PCR	miR-451a, miR-340-5p, miR-136-5p, miR-29b-3p	miR-4292	[118]	2013
22 (TB), 14 (LTBI)	19 HC	Germany	CD4 + T cells	literature (miRNA selection)	qRT-PCR		miR-21-5p, miR-26a-5p, miR-29a-3p, miR-142-3p	[39]	2013
24 (TB)	20 HC	Argentina	PBMCs	literature (miRNA selection)	qRT-PCR	miR-424-5p	miR-146a-5p	[119]	2012
29 (TB), 29 (LTBI)	18 HC	China	PBMCs	Microarray (Agilent)	qRT-PCR	miR-424-5p, miR-365a-3p (vs HC); miR-424-5p, miR-365a-3p, miR-144-3p, miR-223-3p, miR-451a (vs LTB)		[120]	2011
21 (TB)	19 HC	China	PBMCs induced with PPD	Microarray (Agilent)	qRT-PCR	miR-155-5p, miR-155-3p		[112]	2011
